# The Outcomes of “Submitted” Publications From Applicants to Orthopaedic Surgery Residency Programs: A Retrospective Review of 1303 Residency Applications

**DOI:** 10.5435/JAAOSGlobal-D-20-00112

**Published:** 2020-07-20

**Authors:** Ryan D. Freshman, Xavier C. Cortez, Hubert T. Kim, Brian T. Feeley, Alan L. Zhang, Drew A. Lansdown

**Affiliations:** From the Department of Orthopedic Surgery (Dr. Freshman, Dr. Kim, Dr. Feeley, Dr. Zhang, and Dr. Lansdown), University of California San Francisco, San Francisco, CA, and the University of California San Francisco School of Medicine (Mr. Cortez), San Francisco, CA.

## Abstract

**Purpose:**

To evaluate research listed as “Submitted” on orthopaedic surgery residency applications for eventual publication rates and quality.

**Significance:**

As the orthopaedic surgery residency selection process becomes increasingly competitive, the number of research publications listed on applications continually increases. However, the utility of using publications listed as “Submitted” in the applicant evaluation process remains unknown.

**Methods:**

Demographic and publication data were retrospectively collected from 1303 applications to an orthopaedic surgery residency program. The PubMed database was used to verify “Submitted” publications for (1) publication fruition or (2) publication mismatch, defined as discordance between the listed journal of submission and the eventual journal of publication.

**Results:**

A total of 594 applications (45.6%) listed ≥1 publication as “Submitted.” Out of 1636 “Submitted” publications, 565 were unverifiable (32.5%). Of the 1071 verified publications, 362 (33.8%) experienced publication mismatch. Within this subgroup, a significant difference existed between the mean impact factors of the listed journal of submission and the eventual journal of publication (1.5 ± 2.7 versus 3.0 ± 2.5, *P* < 0.01). Demographic data were not predictive of having an unverified publication.

**Conclusion:**

Publications listed as “Submitted” in orthopaedic surgery residency applications frequently remain unpublished or are published in less impactful journals than originally intended.

Gaining admission to an orthopaedic surgery residency is a demanding task. The modern applicant typically pursues multiple elective away rotations in orthopaedic surgery before being selected for a series of interviews, during which they must present themselves as accomplished well-rounded individuals who have a genuine interest in the field of orthopaedic surgery. To achieve success in the residency selection process, applicants must seek to distinguish themselves from their peers through a variety of objective and subjective metrics, including United States Medical Licensing Exam (USMLE) step 1 and step 2 scores, letters of recommendation, and clerkship grades.

One such metric that is commonly used to evaluate applicants is scholarly research activity. The number and quality of research publications listed on an application can provide insight to an applicant's ability to understand the scientific process and carry out a research project from inception to completion. Additionally, the presence of orthopaedic research helps demonstrate an applicant's interest in the field of orthopaedic surgery. Given the increasingly competitive nature of applying to orthopaedic surgery residencies, the quantity of scholarly research disclosed by applicants has increased drastically. From 2007 to 2016, the mean number of research abstracts, presentations, and publications listed on applications to orthopaedic surgery residency programs increased nearly threefold, from 3.0 to 8.2.^1,2^ This statistic consists of both “verified” publications that have been published in print or online by a scientific journal and “submitted” projects that are not yet published or currently under consideration by a scientific journal. Unfortunately, given the competitive nature of matching into orthopaedics, this “submitted” project category presents an opportunity for applicants to overrepresent or misrepresent their research accomplishments to appear as more competitive candidates.

Publication misrepresentation has already been well-studied in multiple fields, including orthopaedics.^3,4,5,6,7,8,9,10,11,12,13,14,15,16^ Rates of misrepresentation in orthopaedics have significantly decreased over the last 20 years, likely due to the advent of including PubMed identification numbers in the Electronic Residency Application Service (ERAS) standardized residency application.^6,10,12^ While previous studies have examined publication misrepresentation among verified publications, no study to date has examined the outcomes of publications listed as “submitted” at the time of application to orthopaedic surgery residency. These “Submitted” publications have no associated PubMed identification, which prevents their outcomes from being verified during the residency application process.

Interpreting the value of these “Submitted” publications therefore remains unclear. Thus, the purpose of this study was to examine a population of orthopaedic surgery residency applicants to characterize the likelihood of eventual publication of research projects listed as “Submitted” at time of application to orthopaedic surgery residency. We also sought to identify applicant demographic factors that may affect the likelihood of eventual publication and compare the impact factor of a publication's listed journal of submission with that of its eventual journal of publication. We hypothesized that the eventual journal of publication would have a significantly lower impact factor than the listed journal of submission.

## Methods

Demographic and research publication data from three application cycles (2013 to 2014 through 2015 to 2016) were retrospectively collected from applications to an orthopaedic surgery residency program at a large academic center. These applications cycles were chosen to provide for sufficient time (>1 year) for “Submitted” publications to be reviewed and published in scientific journals. All applicant data were deidentified after data collection and before data analysis. Demographic data consisted of applicant age, gender, US World and News Report medical school research funding ranking, USMLE step 1 score, USMLE step 2 score (when reported), Alpha Omega Alpha membership, status as American medical graduate (AMG) versus international medical graduate (IMG), the presence of an advanced degree (defined as a Master's degree or PhD), and the presence of additional research time beyond the standard four-year medical school timeline to perform scholarly work (Table 1). Research publication data consisted of the total number of listed publications, the number of “Submitted” publications, the number of discrepancies in “Submitted” publications, and average impact factors of both journals of submission on the ERAS application and eventual journals of publication, as reported in the PubMed database.

**Table 1:** Demographic Characteristics of Orthopaedic Surgery Applicants From 2013 to 2016

Applicant Demographics (n = 1303)	
Age, mean (SD)	27.1 (2.4)
Male, n (%)	1090 (83.7)
Female, n (%)	213 (16.3)
Step 1, mean (SD)	247.6 (9.9)
Step 2, mean (SD)	252.0 (12.7)
AOA, n (%)	377 (28.9)
AMG, n (%)	1288 (98.8)
NIH Top 40 research, n (%)	542 (41.6)
Advanced degree, n (%)	231 (17.7)
Year off, n (%)	185 (14.2)

AMG = American medical graduate, AOA = Alpha Omega Alpha

Both verified and “Submitted” publications were reviewed and tracked using the PubMed database to evaluate for (1) publication fruition and (2) concordance between the journal of submission as listed on the ERAS application and the final accepting journal as identified by the PubMed database. If there was difficulty in accessing an article through a journal's website, an Internet search engine was used. Research conference podium presentations and posters listed as “Submitted,” as well as articles listed as submitted to nonpeer reviewed publications, were excluded from data collection. Verified publications were defined as publications confirmed via the PubMed database to be published in a scientific journal. Unverified publications were defined as those that could not be identified via the PubMed database or Internet search engine or those containing incorrect authorship listings. Publication mismatch was defined as discordance between the listed journal of submission on the ERAS application and the eventual journal of publication as reported in the PubMed database. Impact factors were derived from Journal Citation Reports; unlisted journals were assigned an impact factor of 0. Continuous variables were compared using a 2-tailed Student *t* test for normally distributed data, and categorical variables were compared using a chi-square test. Significance was defined as *P* < 0.05.

## Results

**Figure 1:**
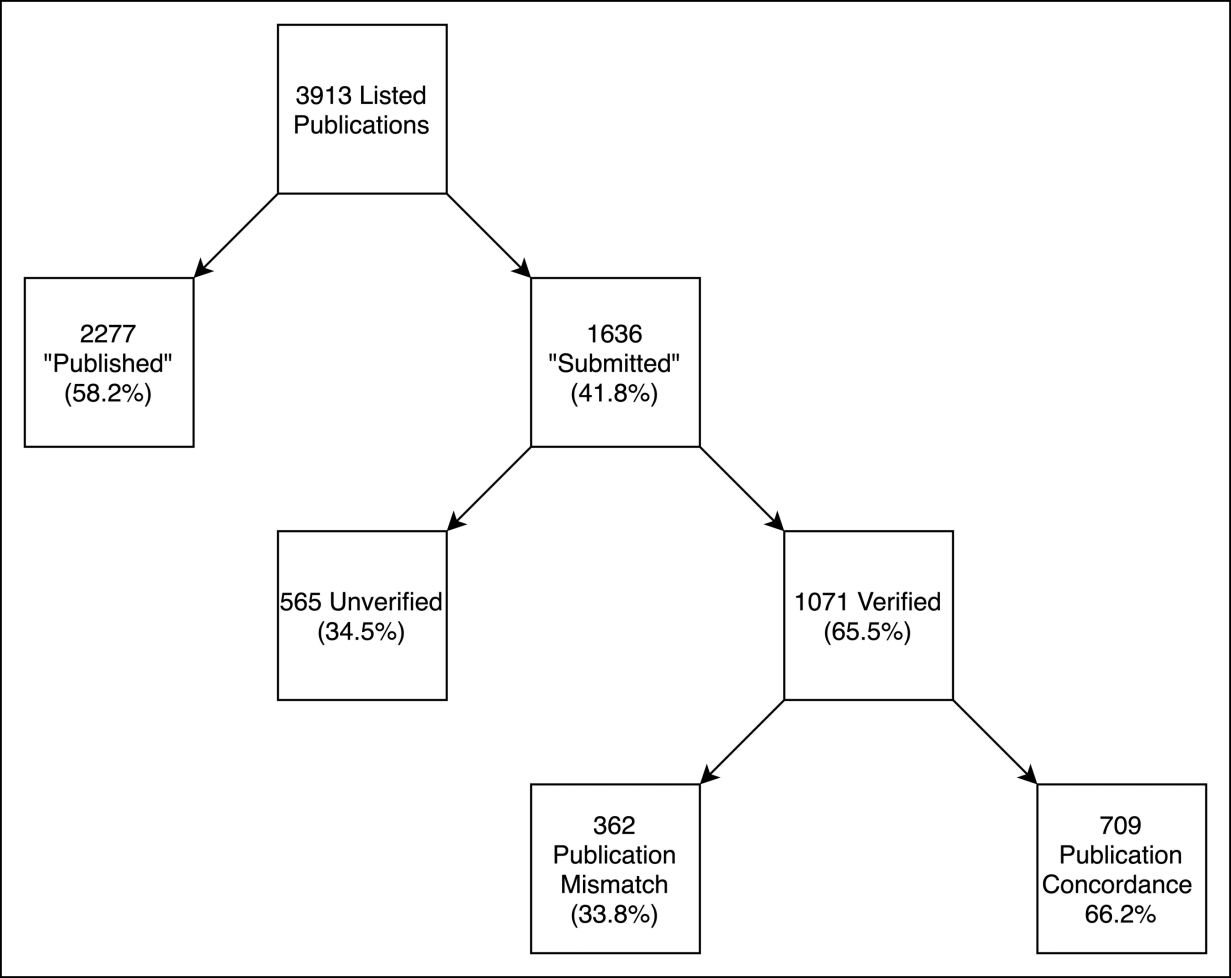
Flowchart showing publication demographics. A total of 3913 publications were listed among 1303 applicants; 594 applicants accounted for the 1636 “Submitted” publications. “Submitted” publications comprised 41.8% of all listed publications.

A total of 1303 residency applications were submitted during the three application cycles; 594 of 1303 applicants (45.6%) listed one or more publications as “Submitted.” The proportion of applicants with one or more “Submitted” publication ranged from 41.1% to 47.2% over the three years of the study, though no significant difference was observed between years (*P* = 0.072). A total of 1636 “Submitted” publications were listed among these 594 applicants, resulting in a mean of 2.75 “Submitted” publications per applicant (Figure 1). The group of “Submitted” publications consisted of 1071 verified publications (65.5%) and 565 unverifiable publications (34.5%). Of the unverified publications, 78.8% could not be identified via PubMed or Internet search engine, while 21.2% had incorrect authorship listings.

When examining relationships between applicant demographic factors and unverified publications, IMGs (n = 6) had significantly more unverified publications than AMGs (n = 588) (2.3 ± 3.9 versus 0.9 ± 1.2, *P* < 0.01, 95% confidence interval [CI] −0.405 to −2.397). Applicants who had taken a year off from their medical training to perform research (n = 136) had a significantly higher number of “Submitted” publications than applicants whose medical training was uninterrupted (n = 458) (3.3 ± 4.0 versus 0.9 ± 1.8, *P* < 0.001, 95% CI 2.053 to 2.756). However, these applicants who had taken a year off from their medical training also had significantly more unverified publications (1.15 ± 1.385 versus 0.89 ± 1.192, *P* = 0.003, 95% CI 0.022 to 0.497). Similarly, applicants from National Institutes of Health (NIH) Top 40 institutions (n = 301) had significantly higher numbers of “Submitted” publications than applicants from non-NIH Top 40 research institutions (n = 293) (1.7 ± 2.8 versus 0.9 ± 1.7, *P* < 0.01, 95% CI 0.551 to 1.076), but no significant difference was seen in rates of unverified publications between the 2 groups (*P* = 0.86).

Univariate analysis of the 594 applicants with at least one “Submitted” publication was performed. Applicant gender, USMLE step 1 and step 2 scores, Alpha Omega Alpha membership, institutional research ranking on NIH Top 40 list, AMG/IMG status, the presence of an advanced degree, and the presence of extending medical school beyond four years for extra research time were not predictive of an applicant having ≥1 unverified publication (Table 2).

**Table 2:** Univariate Analysis of Applicants With ≥1 Publication in Submission

Univariate Analysis of Applicants With ≥1 Publication In Submission (n = 594)			
Variable	≥1 Error (n = 331)	No Errors (n = 263)	*P*
Age, mean (SD)	27.0 (2.2)	27.0 (2.8)	0.214
Gender, n (%)			0.164
Male	289 (87.3)	219 (83.3)	—
Female	42 (12.7)	44 (16.7)	—
Step 1, mean (SD)	248.9 (10.0)	247.7 (10.3)	0.436
AOA, n (%)	86 (26.0)	69 (26.2)	0.944
AMG, n (%)	328 (99.1)	260 (98.9)	1.000
NIH Top 40 research, n (%)	157 (47.4)	144 (54.8)	0.076
Advanced degree, n (%)	57 (17.2)	46 (17.5)	0.931
Year off, n (%)	78 (23.6)	58 (22.1)	0.663

AMG = American medical graduate, AOA = Alpha Omega Alpha

**Figure 2:**
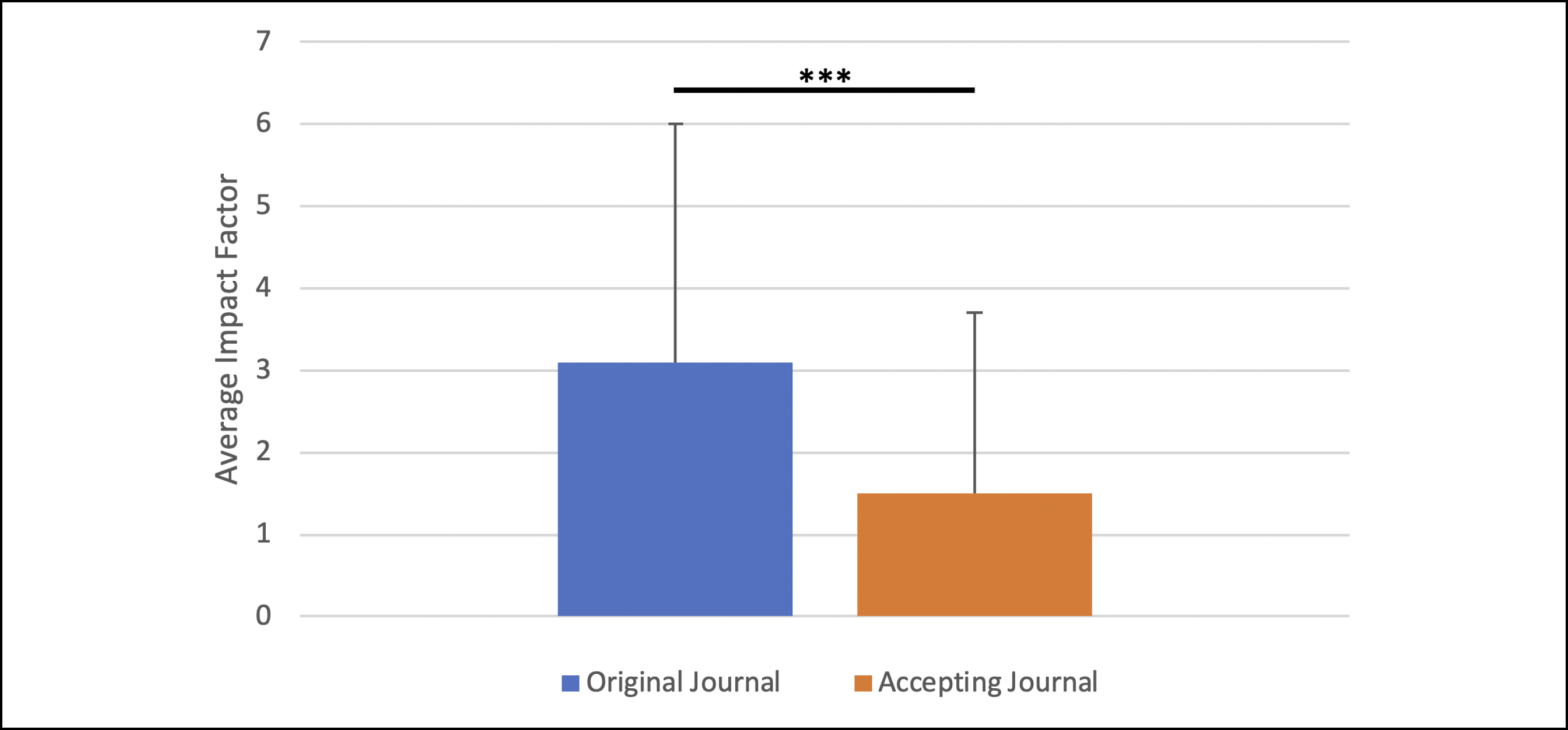
Chart showing the average impact factor of publication ultimately accepted to a different journal. Average impact factor of the original journal of submission (3.0 ± 2.5) was significantly higher than the average impact factor of the accepting journal (1.5 ± 2.7). *** denotes *P*-value < 0.01.

Within the cohort of 1071 verified publications, 362 (33.8%) publications eventually appeared in a different journal than the journal listed on the residency application (Figure 1). Among these publications, the mean impact factor of the eventual journal of publication was significantly lower than the mean impact factors of the journal of submission (1.5 ± 2.2 versus 3.1 ± 2.9, *P* < 0.01; Figure 2).

## Discussion

We observed that “Submitted” publications frequently remain unpublished for at least 2 years after residency application submission. Our original hypothesis regarding publications experiencing publication mismatch was validated because we discovered that the eventual journal of publication had a significantly lower impact factor than the listed journal of submission. Finally, IMGs had more inaccurate or unverified research listings compared to AMGs, but no other demographic factors were associated with increased likelihood of an applicant having an inaccurate or unverified research listing.

In our study, 35% of “Submitted” publications were not successfully published, despite at least 3 years of time, since the final included application cycle. This observation would support program directors and faculty interviewers placing less weight on “Submitted” publications when reviewing residency applications. Rates of successful publication among “Submitted” research publications have been previously studied in residency applicants from nonorthopaedic fields. Approximately half of all publications listed as “Submitted” in urology and radiology residency applications were successfully published after 1 and 2 years, respectively.^14,17^ Our study reported a higher rate of successful publication (65.5%) although our results likely differed from these prior studies because our publication outcomes were tracked for a minimum of 3 years after residency application submission. The successful publication rate reported in our findings mirrors that from Okike et al,^18^ who found similar rates of successful publication at 3 years after a rejection from the *Journal of Bone and Joint Surgery*. While these results do not serve as a direct comparison to our study, they provide a good approximation of the time required for a “Submitted” publication to reach publication in a scientific journal. Additionally, the publication of research from residency applicants may be even more of a challenge as applicants switch institutions. This rate of persistently unpublished work should also prompt program directors to continue to encourage their new trainees to complete prior scholarly work.

We did identify that the likelihood of having an unverified publication was significantly higher for applicants with a greater number of “Submitted” publications. Additionally, applicants from NIH Top 40 research institutions will likely have more “Submitted” publications as their home institutions are shown to have higher levels of scholarly research activity. However, it is unclear why IMGs had significantly more unverified publications than AMGs. The literature surrounding this topic is inconclusive, as some studies have shown IMGs to have higher rates of research misrepresentation, whereas others have found no differences from AMGs in fields outside of orthopaedic surgery.^3,11,19^ There may be several factors contributing to our results. IMG match rates into orthopaedic surgery have historically been well below AMG match rates,^20^ and this knowledge may influence IMGs to pursue additional scholarly research to bolster their application, which in turn increases their chance of having unverified publications. Additionally, IMGs have been shown to be held to a higher standard than AMGs during the residency screening process,^21^ which may further contribute to a perceived need for increased research output that leads to more unverified publications. However, given the few numbers of IMGs in this study, the possibility is that these results were driven by sample size alone and that further investigation with larger sample sizes is warranted.

Publication mismatch was moderately prevalent within our study because 33.8% of “Submitted” publications experienced publication mismatch with eventual publication in a less impactful journal than originally intended. Though rates of publication mismatch range from 41% to 49.2% in the nonorthopaedic literature,^14,17^ our findings regarding impact factor are in line with these prior studies, which found that the impact factor of the final journal of publication is significantly lower than the original journal of submission. Despite the prevalence of publication mismatch, it is difficult to know if it is driven by applicants' willful intent to gain a competitive advantage through deception or by factors beyond their control. It has been previously shown that applicants who list multiple unpublished manuscripts to inflate their resume have an increased chance of matching into a competitive specialty^22^; thus, it is possible that applicants list multiple unpublished publications as “Submitted” to appear more favorably to residency selection committees. However, given that successful publication rates in high-impact orthopaedic surgery journals can be as low as 20%^23^ and that applicants are typically not the primary decision-makers when choosing a journal of submission, it is also possible that publication mismatch occurs as an almost inevitable aspect of the research publication process and does not represent applicant deceit. Additional studies into publication mismatch and the authors' motivations behind selecting journals of submission may help further our understanding of this topic.

As increasing amounts of research publications are being listed on ERAS applications, the results of our study will be useful to program directors and others involved in the residency selection process as they select candidates for interview and final rank list. In the future, research publications may play a larger role in these selection processes given current plans to transition USMLE step 1 results from a scaled scoring system to a pass/fail system. Given the significant portion of self-reported “Submitted” publications that remained unverifiable in our study, the number of “Submitted” publications does not currently appear to be a reliable measure of an applicant's research productivity. However, modifications made at several points in the residency application process would improve the utility of using “Submitted” publications to evaluate applicants and provide residency selection committees with valuable data to aid in their decision-making. The online ERAS application could be adjusted to allow for real-time updates with accepted, rejected, or resubmitted publications to allow applicants to accurately represent of their research status at the time of residency interviews. Applicants could also be required to provide a proof of submission for all “Submitted” publications, or journals of submission could be required to assign a reportable identification number similar to a PubMed ID to each “Submitted” publication. Finally, in addition to these proposed changes, interviewers should also take time to clarify the current status of all works listed as “Submitted.” This step is especially important given the three- to 5-month lag time between when applicants submit their residency applications and when they participate in in-person interviews for orthopaedic surgery residency programs.

Our study was able to follow up on a large sample of “Submitted” publications from orthopaedic surgery applicants and define the rates of successful publication and publication mismatch. However, our study also had several limitations. First, our study was a retrospective analysis of applications, which limits the conclusions we can draw from our results. Second, this study was performed at a single academic institution and may represent a biased study population, which limits the generalizability of our results. Third, it is possible that some unverified publications were actually published in journals not accessible through PubMed, Medline, or Google Scholar and thus were not verifiable using our methodology.

## Conclusions

“Submitted” publications listed on orthopaedic surgery residency applications frequently remain unpublished or published in less impactful journals than originally intended. This phenomenon is likely multifactorial in origin. Given the current uncertainty surrounding such publications, they are not good predictors of an applicant's research productivity. However, changes to the application process that allow for reliable reporting and verification of “Submitted” publications may help to clarify their impact and improve upon the decision-making framework of future residency selection committees.
